# The prevalence of obstructive sleep apnea-hypopnea syndrome in patients with cystic fibrosis: An updated systematic review and meta-analysismeta-analysis

**DOI:** 10.1097/MD.0000000000049828

**Published:** 2026-07-17

**Authors:** Jun Zeng, Hua Yu, Jia Liu, Jiaqing Jiang, Jia Li, Jie He

**Affiliations:** a School of Clinical Medicine, Chengdu Medical College, Chengdu, Sichuan, China; b Department of Pulmonary and Critical Care Medicine, The First Affiliated Hospital of Chengdu Medical College, Chengdu, Sichuan, China; c Key Laboratory of Geriatric Respiratory Diseases of Sichuan Higher Education Institutes, Chengdu, Sichuan, China; d Orthopedics Department, The First Affiliated Hospital of Chengdu Medical College, Chengdu, Sichuan, China; e General Practice Department, The First Affiliated Hospital of Chengdu Medical College, Chengdu, Sichuan, China.

**Keywords:** cystic fibrosis, OSAHS, prevalence, PSG, pulmonary function

## Abstract

**Background:**

This research aimed to evaluate the prevalence of obstructive sleep apnea-hypopnea syndrome (OSAHS) in individuals with cystic fibrosis (CF) and to examine its relationship with sleep monitoring parameters and pulmonary function.

**Methods:**

Five databases (EMBASE, PubMed, Web of Science, CNKI, and Cochrane Library) were searched for publications on the prevalence of OSAHS in CF. Two independent researchers assessed study quality using the Joanna Briggs Institute tool. Weighted mean differences were calculated for continuous variables, and the model choice (random vs fixed effects) was determined by whether *I*^2^ exceeded 50%.

**Results:**

Fourteen studies were included: 11 in the prevalence meta-analysis and 13 in the mean apnea-hypopnea index (AHI) analysis. Six studies compared sleep and pulmonary parameters between CF patients with and without OSAHS. The pooled prevalence of OSAHS in CF was 48% (*I*^2^ = 96.5%), slightly higher in pediatric than adult patients. The mean AHI was 3.47 in pediatric patients with CF and 6.19 in adult individuals with CF. Compared to OSAHS-negative patients, OSAHS-positive patients exhibited higher AHI and arousal index, lower mean and minimum oxygen saturation, and worse pulmonary function.

**Conclusion:**

There is a significant prevalence of OSAHS among patients with CF, notably within the pediatric demographic. Patients with CF generally present with elevated AHI values and poorer pulmonary function in those with concurrent OSAHS. The findings advocate for earlier polysomnography screening in patients with CF to facilitate timely intervention.

## 1. Introduction

Cystic fibrosis (CF) is an autosomal recessive multisystem disorder instigated by mutations in the CF transmembrane conductance regulator (CFTR) gene, resulting in severe chronic airway disease, even in childhood. This results in the gradual decline of pulmonary function, frequent hospitalizations for treatment, and early mortality.^[1,2]^ Patients with CF experience diminished sleep quality and more frequent awakenings caused by persistent coughing, snoring, and sleep-disordered breathing, including hypoxemia and obstructive sleep apnea-hypopnea syndrome (OSAHS), thereby reducing sleep efficiency.^[3–5]^ As highlighted by various epidemiologic studies, OSAHS is quite common, with a prevalence of 24% in men and 9% in women aged 30 to 60. It can appear at any age in the pediatric population, from neonatal to adolescence, with an estimated prevalence between 2 and 5.7%.^[6–8]^ However, in patients with CF of the same age group, this prevalence is markedly higher, affecting over 50% of the population, and may vary depending on the apnea-hypopnea index (AHI) threshold employed to characterize obstructive sleep apnea syndrome (OSAS) in each study.^[1,9]^ Recent research highlights that in adult patients with CF, the prevalence of OSAHS reaches up to 69%.^[10]^ Besides identifying the high prevalence of OSAHS in patients with CF, prolonged episodes of hypoxemia during sleep have also been noted in adult patients with CF.^[11–14]^ OSAHS might affect the quality of life of individuals with CF.

Significant advancements in the care of patients with CF over time have potentially impacted the prevalence of respiratory disorders during sleep. The introduction and utilization of CFTR modulators have shown promise in substantially enhancing pulmonary health in patients with CF,^[15–17]^ which may consequently reduce the frequency or severity of OSAHS. With the advent of effective treatments for CF, adults with CF are now experiencing longer life expectancies,^[18]^ and their body weight has progressively increased (attributed to improved nutritional management and weight gain from new CFTR modulators).^[19]^ Age progression and elevated body mass are significant risk determinants for OSAHS. As a result, the current landscape of respiratory disorders during sleep in adults with CF might have evolved, necessitating a deeper understanding of its present epidemiology. A previous systematic review and meta-analysis^[3]^ demonstrated that people with CF exhibit reduced AHI values, reduced lowest peripheral oxygen saturation (SpO_2_) during sleep, diminished sleep efficiency, and a decreased proportion of total rapid eye movement (REM) sleep duration relative to healthy age-matched controls. However, that study did not address the prevalence of OSAHS, the correlation between OSAHS and pulmonary function, or the influence of OSAHS and its management on patients with CF, all of which warrant further investigation.

Given the rising life expectancy of patients with CF, screening for OSAHS via polysomnography (PSG) has become essential for mitigating disease advancement and improving the quality of life in patients with CF. To accomplish this, a precise understanding of OSAHS prevalence must first be established. Thus, this systematic review and meta-analysis aimed to ascertain the global prevalence of OSAHS in adult and pediatric individuals with CF and compare the disparities in sleep monitoring parameters and pulmonary function between patients with CF with and without OSAHS.

## 2. Methodologies

The International Prospective Register of Systematic Reviews documented the systematic review protocol (ID: CRD42024556089) and adhered to the Meta-analysis of Observational Studies in Epidemiology guidelines.^[20]^

### 2.1. Ethics and dissemination

Given the retrospective nature of the current study, the requirement for ethics approval was waived.

### 2.2. Data sources and search strategy

A comprehensive literature search was executed on February 6, 2025, utilizing 5 electronic databases (PubMed, EMBASE, Web of Science, CNKI, and Cochrane Library). Table S1, Supplemental Digital Content 1 provides a detailed description of the research question and search keywords. Publications prior to the identification of the CFTR gene in 1989 were omitted.

### 2.3. Inclusion and exclusion criteria

The Population, Exposure, Comparison, Outcome framework was employed to establish the literature selection criteria: studies involving pediatric (age < 18 years) and/or adult (age ≥ 18 years) individuals with CF were included, regardless of the presence of complications. The primary outcome was the prevalence of OSAHS (AHI ≥ 1/hour in pediatric patients and AHI ≥ 5/hour in adults).^[21]^ Secondary outcomes included the AHI in patients with CF, AHI, arousal index, mean oxygen saturation, minimum oxygen saturation, forced expiratory volume in 1 second (FEV1), % predicted, and forced vital capacity (FVC), % predicted in CF patients with OSAHS and CF patients.

Studies encompassing randomized or non-randomized controlled trials and cohort studies were incorporated. The following criteria were used for exclusion: case reports and case series with fewer than 10 subjects, and studies without original data, were excluded; research involving subjects with diagnoses of conditions distinct from CF was omitted; research involving subjects with diagnoses of conditions distinct from OSAHS was omitted; personal reports, letters, conference abstracts, and animal experiments were excluded. In case of duplication, only the study with a large sample size and complete information was selected.

After importing the literature into EndNote X9.3.3 reference management software, duplicates were eliminated automatically and manually. Two independent researchers assessed the titles, abstracts, and full texts based on predefined criteria. Any disagreements encountered during the screening process were resolved with the assistance of a third researcher. In instances of overlap in the included populations, the study with the higher participant count was preferentially included in this review.

### 2.4. Data extraction and quality assessment of included studies

Two researchers autonomously collected data and documented it in a standardized data collection form. The data collected for each study included: title, first author, year of publication, country, study design type, and total number of study participants. Additionally, demographic and clinical characteristics (age, gender, body mass index (BMI), genotype, FEV1, % predicted, FVC % predicted) of OSAHS patients within the study population were also documented. Table 1 presents the study data utilized for evaluating each outcome. Two authors evaluated the quality of the studies utilizing the Joanna Briggs Institute quality assessment tool. Specifically, 8 criteria were assessed: inclusion criteria and sample definition, comprehensive description of the study population, validation and reliability in exposure measurement, standards and objectives for measuring conditions, identification of confounding variables, confounding factor strategies, validity and reliability of outcomes, and appropriate statistical analysis in all included studies. One point was allocated for each criterion: studies scoring 7 to 8 were deemed high quality, 4 to 6 were moderate quality, and studies scoring 0 to 3 were low quality.^[20]^ Any disputes arising during the data extraction or quality assessment process were resolved through negotiation.

**Table 1:** Characteristics of eligible studies included in the systematic review.

Author	Year	Country	Population	Sample (n)	Age (yrs)	Sex Male (%)	FVC (%)	FEV1 (%)	BMI (kg/m^2^)	SK score	AHI (n/h)	Mean SpO_2_ (%)	Minimum SpO_2_ (%)	Arousal index	Research type	Referral population (with symptoms)	Study design
Maqsood A	2024	Ireland	Adults	42	34.7 (18.9–60.6)	66.7	NA	62.5	24.2	NA	8.90 ± 9.70 (NREM)	92.30 ± 2.50	86.40 ± 4.70	NA	Retro	Yes	CS
								(18.9–93.5)	(17.8–38.3)		25.90 ± 22.60 (REM)						
Shakkottai A	2024	USA	Children	49	8.63 ± 5.11	53.06	NA	91.63 ± 20.34	NA	NA	2.90 ± 4.00	96.50 ± 1.30	88.80 ± 5.10	11.00 ± 4.40	Retro	Yes	CSS
Vezir D	2023	Turkey	Adults	35	30.10 ± 13.10	60	69.70 ± 20.30	59.40 ± 24.50	23.10 ± 4.40	NA	5.86 ± 5.64	92.1	87	NA	Pro	No	CSS
												(90.1–94.4)	(82.0–91.0)				
Shakkottai A	2022	USA	Adults	32	35.00 ± 12.50	47	NA	61.40 ± 28.70	24.60 ± 5.30	NA	10.20 ± 5.30	94.40 ± 2.40	87.30 ± 4.10	15.70 ± 7.60	Retro	Yes	CS
(Adults)																	
Shakkottai A	2022	USA	Children	42	8.90 ± 05.00	60	NA	91.60 ± 18.30	NA	NA	10.20 ± 5.30	94.40 ± 2.40	87.30 ± 4.10	15.70 ± 7.60	Retro	Yes	CS
(Children)																	
Welsner M	2022	Germany	Adults	52	30.70 ± 8.00	65	66.40 ± 17.90	49.10 ± 14.80	21.50 ± 3.30	NA	6.00 ± 3.80 (NREM)	91.00 ± 2.80	83.80 ± 3.60	22.40 ± 9.80	Pro	No	CS
											18.30 ± 11.20 (REM)						
Barbosa RRB	2020	Brazil	Children	31	9.6	35.5	61.80 ± 21.50	52.40 ± 28.60	16.60 ± 2.60	90	2.46 ± 2.02	95	88.5	8.8 (6.7–11.2)	Pro	No	CSS
					(7.9–15.1)					(75.0–95.0)		(94.0–97.0)	(83.0–91.0)				
Isaiah A	2019	USA	Children	35	11.6	57	NA	60.7	42.1	NA	1.60 ± 1.08	92.9	86.5	10.5 (8.6–12.4)	Retro	Yes	CCS
					(9.5–13.1)			(53.0–68.5)	(31.5–52.6)			(91.7–94.1)	(84.7–88.1)				
Lumertz MS	2019	Brazil	Children	16	11.00 ± 5.60	75	92.60 ± 22.16	86.23 ± 27.24	0.13 ± 0.61	89.51 ± 8.57	2.10 ± 2.00	95.50 ± 2.80	87.60 ± 3.70	NA	Retro	Yes	CSS
									(Z score)								
Waters KA	2017	Australia	Children	46	11.10 ± 1.50	33	87.40 ± 16.50	74.60 ± 18.80	17.90 ± 4.10	NA	NA	96.20 ± 1.80	91.40 ± 3.30	8.70 ± 3.20	Pro	No	CSS
Veronezi J	2015	Brazil	Mixed	34	15.90 ± 7.00	58.82	NA	70.90 ± 30.90	18.30 ± 2.40	72.50 ± 12.80	4.80 ± 2.60	94.80 ± 2.20	86.00 ± 4.90	NA	Pro	No	CSS
Perin C	2012	Brazil	Adults	51	25.1 ± 6.7	47.06	69.84 ± 23.25	57.7 ± 24.7	25.1 ± 6.7	70.61 ± 12.11	0.3 (0–0.5)	92.0 ± 3.5	85.8 ± 6.3	12.1 ± 6.1	Pro	No	CS
Spicuzza L	2012	Italy	Children	40	5.27 ± 2.81	58	82.50 ± 32.85	76.60 ± 42.00	17.80 ± 21.50	NA	9.20 ± 8.10	94.10 ± 5.90	86.20 ± 49.00 (NREM)	NA	Retro	No	CCS
													85.10 ± 38.20 (REM)			No	
Ramos RT	2011	Brazil	Children	67	8	56.7	NA	78.5	NA	85.60 ± 9.10	5.00 ± 3.08	NA	80.00 ± 5.00	7.00 ± 3.08	Retro	Yes	CSS
					(5–10)			(67–92.8)									
de Castro-Silva C	2009	Brazil	Mixed	40	12.93 ± 6.13	65	79.17 ± 20.49	73.92 ± 26.53	18.04 ± 3.49	71.86 ± 19.22	2.11 ± 2.57	93.4 ± 3.20	86.13 ± 7.06	8.69 ± 4.03	Retro	No	CS

AHI = apnea-hypopnea index, BMI = body mass index, CCS = case-control study, CF = cystic fibrosis, CI = confidence interval, CS = cohort study, CSS = cross-sectional study, FEV1 = forced expiratory volume in 1 second, FVC = forced vital capacity, NA = not applicable, NREM =non-rapid eye movement, OSAHS = obstructive sleep apnea-hypopnea syndrome, Pro = prospective, REM = rapid eye movement, Retro = retrospective, SD = standard deviation, SK = Shwachman-Kulczycki, SpO_2_ = peripheral oxygen saturation, USA = United States of America.

### 2.5. Statistical analysis

Stata software (version 11.0; StataCorp LLC) and RevMan 5.3 (Review Manager [RevMan, Computer program]). Version 5.3. Copenhagen: The Nordic Cochrane Centre, The Cochrane Collaboration, 2014) were employed to compile and analyze the retrieved data. A meta-analysis was conducted to identify the prevalence of OSAHS in patients with CF. The overall prevalence of OSAHS and its 95% confidence interval (CI) were estimated using a random-effects model. Heterogeneity was evaluated using the *Q* test and quantified by the *I*^2^ statistic, with *I*^2^ < 40% indicating low heterogeneity, 40 to 60% as moderate, and > 60% as high heterogeneity.^[22]^ A subgroup analysis based on age was performed to assess the impact of age on OSAHS. When comparing differences in sleep and pulmonary function parameters between CF patients with and without OSAHS, weighted mean differences (MD) were utilized for the meta-analysis. For the sensitivity analysis, which examined the effect of individual studies on the pooled effect size, 1 study was excluded at a time. The publication bias of the included studies (≥ 10 studies) was assessed utilizing Begg and Egger tests on Stata 11.0.

## 3. Results

### 3.1. Literature selection

**Figure 1.**
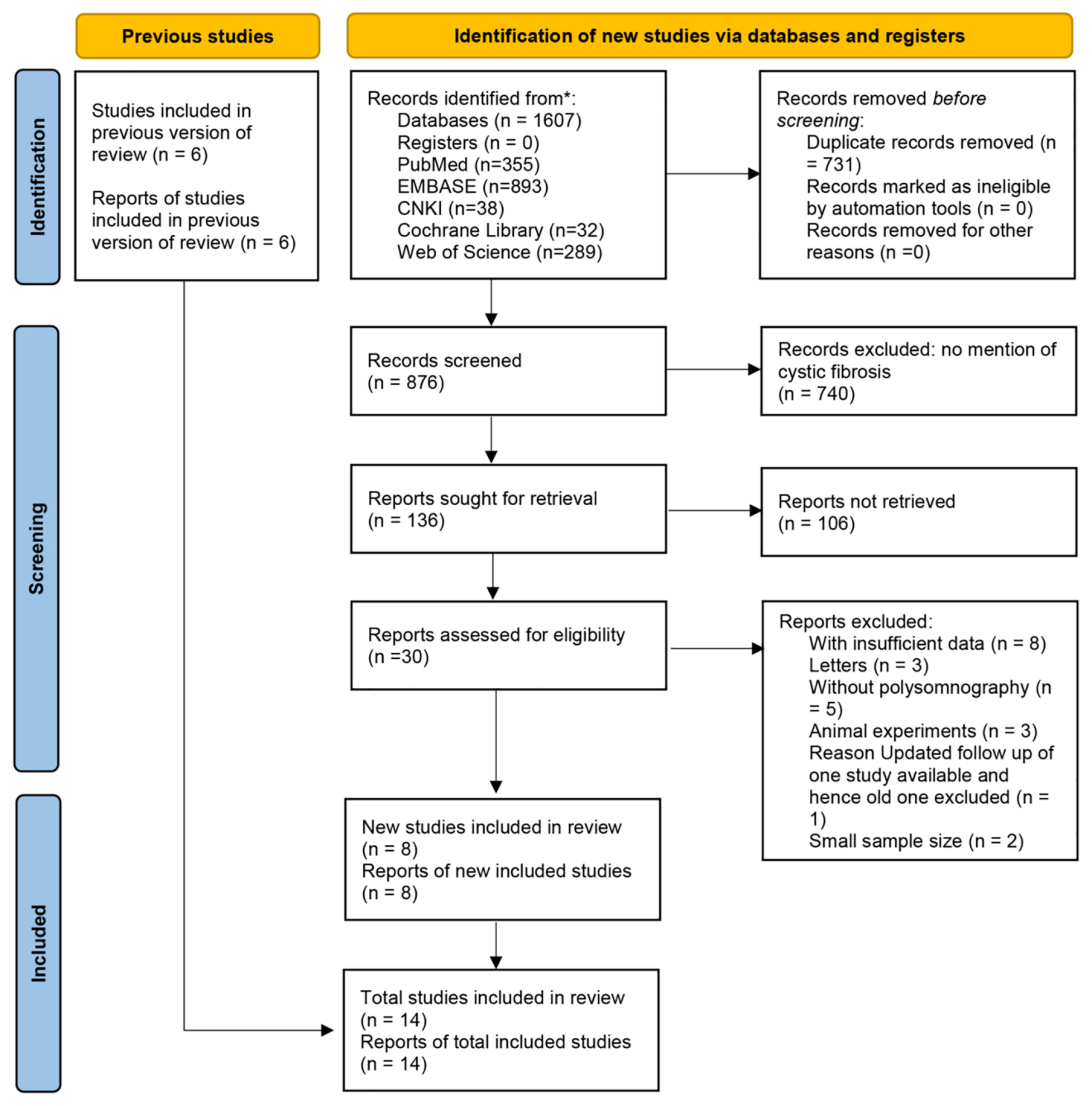
Flow diagram of the literature screening process. n = number of reports.

1607 articles were determined through searching multiple databases (PubMed: 355, Web of Science: 289, EMBASE: 893, CNKI: 38, Cochrane Library: 32). After removing 731 duplicates, 876 articles remained for selection. Title screening excluded 740 articles that were not pertinent to individuals with CF. The residual 136 abstracts were reviewed, and 106 failed to satisfy the inclusion criteria. After obtaining and examining the complete texts of 30 articles, 14 fulfilled the criteria and were incorporated into this systematic review. Of these, 11 were included in the prevalence, and 6 compared sleep monitoring indicators and pulmonary function between OSAHS-positive and OSAHS-negative patients. Figure 1 depicts the study flow chart and grounds for exclusion at each phase.

### 3.2. Literature description

The research incorporated in this systematic review was conducted in pediatric hospitals and CF research centers across various countries, including 6 studies from Brazil, 4 from the United States, and 1 each from Ireland, Turkey, Germany, Italy, and Australia.^[5,9,10,23–33]^ These studies were performed between 2009 and 2024. Most of the research utilized a cross-sectional design,^[5,9,26,27,29,32,33]^ with 8 being retrospective.^[9,10,23,28–30,33,34]^ This study included a total of 612 patients with CF. The number of participants varied from 16 to 74 individuals, with a predominance of male participants.

Thirteen studies reported the FEV1, % predicted of the subjects. The FVC, % predicted was documented in 6 studies. The mean BMI values reported in 11 studies ranged from 16.6 to 42.1 kg/m^2^. The mean Shwachman–Kulczycki scores, reported in 4 studies, varied from 70.61 to 90. Regarding PSG data, the mean AHI varied from 0.3 to 25.9 events per hour, the mean SpO_2_ was 91 to 96.5%, and the lowest oxygen saturation ranged from 80 to 91.4%. Table 1 presents the main features of the research incorporated in this review.

### 3.3. Assessment of methodological quality

Table S2, Supplemental Digital Content 2 displays the risk of bias in the included studies. In all cases, the inquiries concerning the study population details, exposure verification reliability, and publication findings were answered affirmatively. Some studies demonstrated high methodological quality, scoring between 7 and 8 points, while others were classified as medium quality, scoring between 4 and 6 points.

### 3.4. Meta-analysis

#### 3.4.1. Prevalence of OSAHS

**Figure 2.**
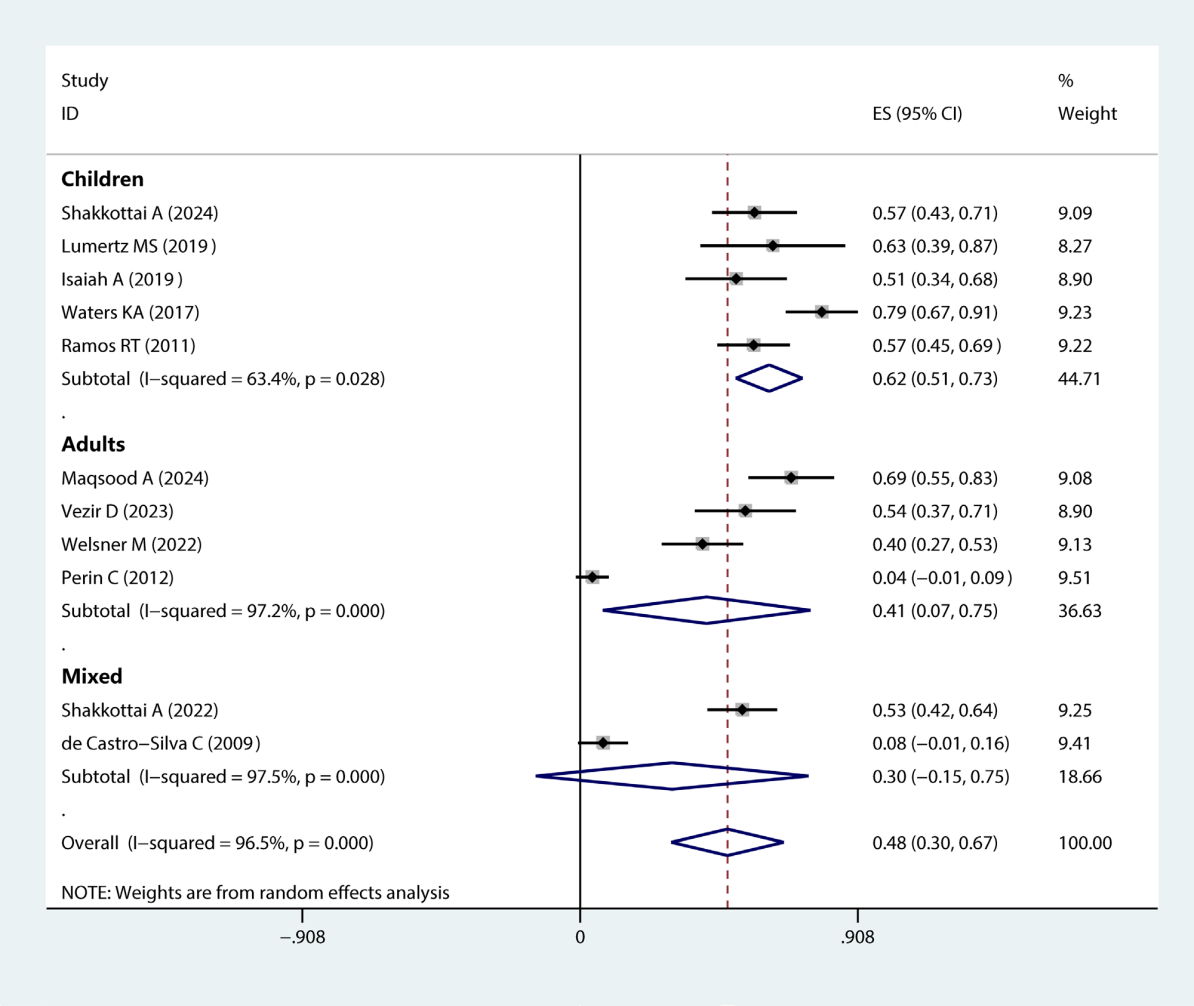
The prevalence of OSAHS in individuals with CF. CF = cystic fibrosis, CI = confidence interval, ES = effect size, OSAHS = obstructive sleep apnea-hypopnea syndrome.

Eleven studies provided data on the prevalence of OSAHS in individuals with CF. The overall prevalence of OSAHS in these individuals ranged from 4 to 79%. After pooling the results utilizing a random-effects model, the analysis indicated that the prevalence of OSAHS in individuals with CF was 48% (95% CI = 0.30–0.67, prediction interval = 0.05–0.93, *I*^2^ = 96.5%, *P* < .001) (Fig. 2). In the prospective study subgroup, the pooled prevalence of OSAHS was 44% (Table 2). An age-based subgroup analysis was carried out to assess the impact of age on prevalence. The findings suggested that the prevalence of OSAHS in pediatric individuals with CF was 62% (95% CI = 0.51–0.73, *I*^2^ = 63.4%, *P* < .001), while in adult patients with CF, it was 41% (95% CI = 0.07–0.75, I^2^ = 97.2%, *P* = .017). Notably, Barbosa et al^[35]^and Spicuzza et al^[24]^ utilized an AHI cutoff point of ≥ 2 for diagnosing OSAHS in pediatrics, reporting prevalence rates of 32% and 73%, respectively, in pediatric patients with CF. The subgroup analyses are shown in Table S1, Supplemental Digital Content 1.

**Table 2:** Subgroup analyses of obstructive sleep apnea in different conditions.

Subgroup analysis of obstructive sleep apnea (n)	ES (95% CI)	*P* Value	*I*^2^ (%)	*P*_h_
Overall (11)	0.48 (0.30–0.67)	< .001	96.5	< .001
Age				
Adult (5)	0.62 (0.51–0.73)	.028	63.4	< .001
Children (4)	0.41 (0.07–0.75)	< .001	97.2	.017
Mixed (2)	0.30 (−0.15–0.75)	< .001	97.5	.186
Research type				
Retrospective (7)	0.51 (0.31–0.71)	< .001	94.1	< .001
Prospective (4)	0.44 (0.05–0.83)	< .001	98.1	.026
Referral population (with symptoms)				
Yes (6)	0.58 (0.52–0.63)	.547	0	< .001
No (5)	0.36 (0.09–0.64)	< .001	97.6	.010
Study design				
CS (5)	0.62 (0.52–0.73)	.040	60.1	<.001
CSS (5)	0.34 (0.10–0.58)	< .001	96.9	.005
CCS (1)	0.51 (0.34–0.68)	NA	NA	< .001

CCS = case-control study, CI = confidence interval, CS = cohort study, CSS = cross-sectional study, ES = effect size, n = number of studies, NA = not applicable, *P*_h_ = *P* for heterogeneity.

##### 3.4.1.1. Publication bias and sensitivity analysis for the meta-analysis of prevalence

Figure S1, Supplemental Digital Content 3 displays the contour-enhanced funnel plot employed to evaluate publication bias. The funnel plot displayed a symmetrical pattern, indicating an absence of publication bias in the meta-analysis (Fig. S1A, Supplemental Digital Content 3). This finding was corroborated by Egger regression test (*P* = .251). The sensitivity analysis suggested that excluding any individual study did not alter the overall findings of the pooled analysis (Fig. S1B, Supplemental Digital Content 3).

#### 3.4.2. AHI in patients with CF

**Figure 3.**
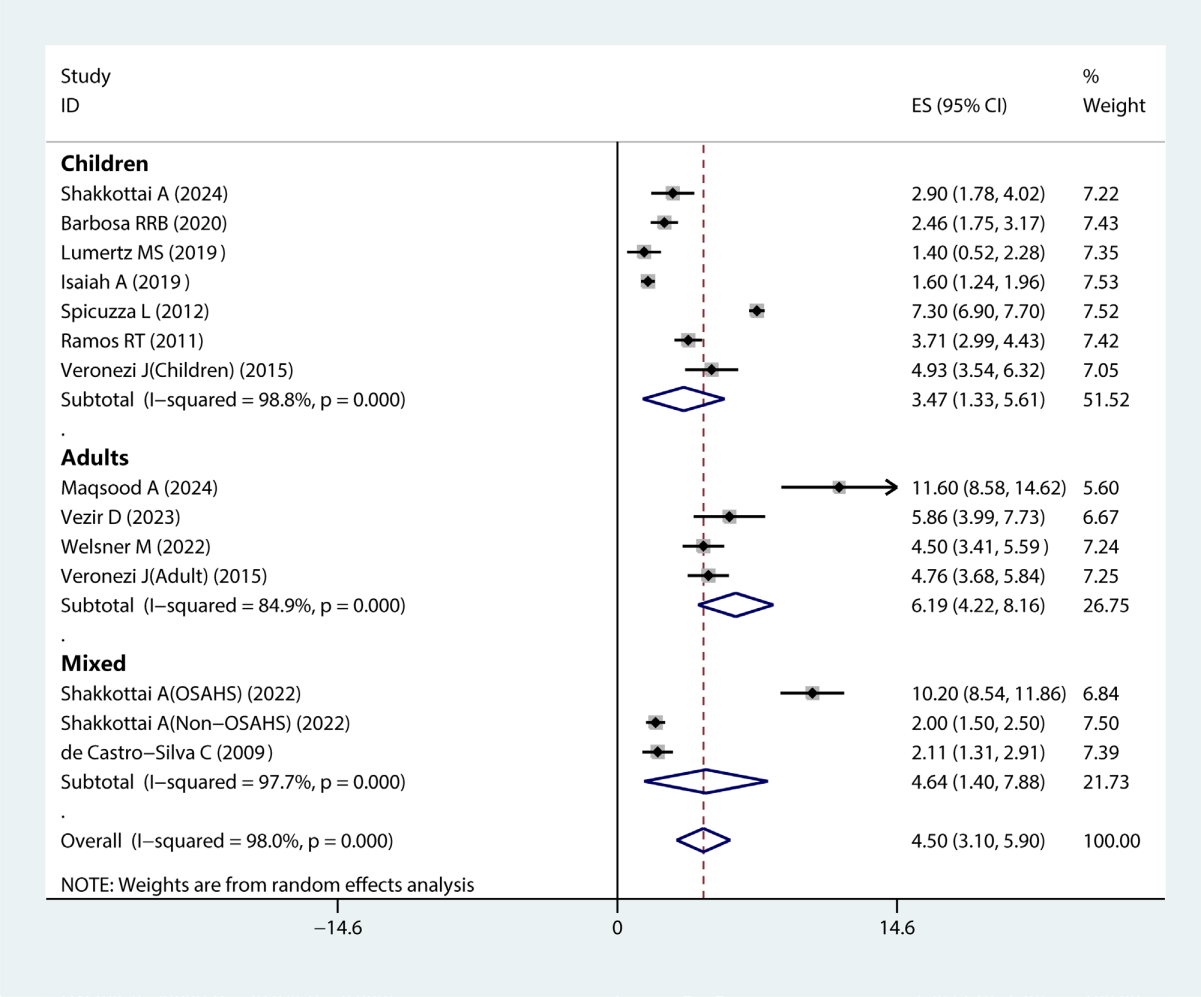
The mean AHI value overnight in patients with CF. AHI = apnea-hypopnea index, CF = cystic fibrosis, CI = confidence interval, ES = effect size.

**Figure 4.**
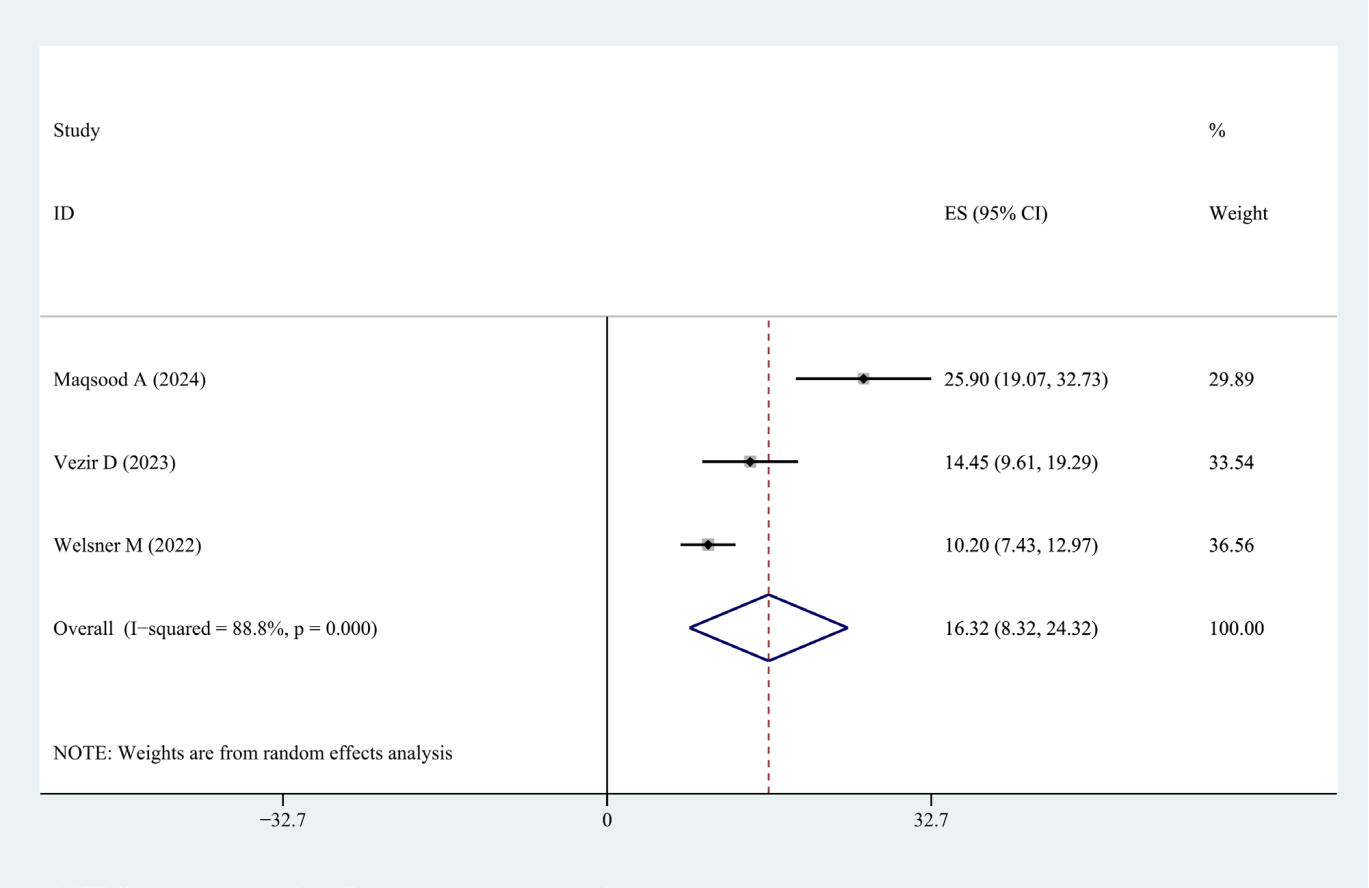
The mean AHI value during rapid eye movement sleep in patients with CF. AHI = apnea-hypopnea index, CF = cystic fibrosis, CI = confidence interval, IV = instrumental variable, OSAHS = obstructive sleep apnea-hypopnea syndrome, SD = standard deviation, SpO_2_ = peripheral oxygen saturation.

Thirteen studies provided mean AHI values for patients with CF, ranging from 1.4 to 11.6. Utilizing a random-effects model, the combined analysis indicated that the mean AHI value of patients with CF was 4.50 (95% CI = 3.10–5.90, *I*^2^ = 98%, *P* < .001) (Fig. 3). A subgroup analysis was conducted to account for the influence of age on AHI. The results showed that the mean AHI for pediatric patients with CF was 3.47 (95% CI = 1.33–5.61, *I*^2^ = 98.8%, *P* < .001), while the mean AHI for adult patients with CF was 6.19 (95% CI = 4.22–8.16, *I*^2^ = 84.9%, *P* < .001). Three studies did not specify age groups, resulting in a combined mean AHI of 4.64 (95% CI = 1.40–7.88, *I*^2^ = 97.7%, *P* = .0051). Three studies reported mean AHI values for patients with CF during the REM stage, and the combined analysis suggested a mean AHI of 16.32 (95% CI = 8.32–24.32, *I*^2^ = 88.8%, *P* < .001) (Fig. 4). The obtained mean AHI values are possibly inflated by the inclusion of referrals (with symptoms) instead of the general population. A subgroup analysis was conducted to account for the influence of population on AHI. The results showed that the mean AHI for the referral population was 4.26 (95% CI = 2.78–5.74, *I*^2^ = 96.2%, *P* < .001), while the mean AHI for the general population was 4.54 (95% CI = 2.59–6.50, *I*^2^ = 97.3%, *P* < .001).

##### 3.4.2.1. Publication bias and sensitivity analysis for the pooled analysis of AHI

Figure S2, Supplemental Digital Content 4 presents the contour-enhanced funnel plot for assessing publication bias. The funnel plot displayed a symmetrical distribution, indicating an absence of publication bias (Fig. S2A, Supplemental Digital Content 4). This was verified by Egger regression test (*P* = .386). Sensitivity analysis suggested that excluding any individual study did not impact the overall results of the pooled analysis (Fig. S2B, Supplemental Digital Content 4).

#### 3.4.3. Impact of OSAHS on sleep indexes in patients with CF

**Figure 5.**
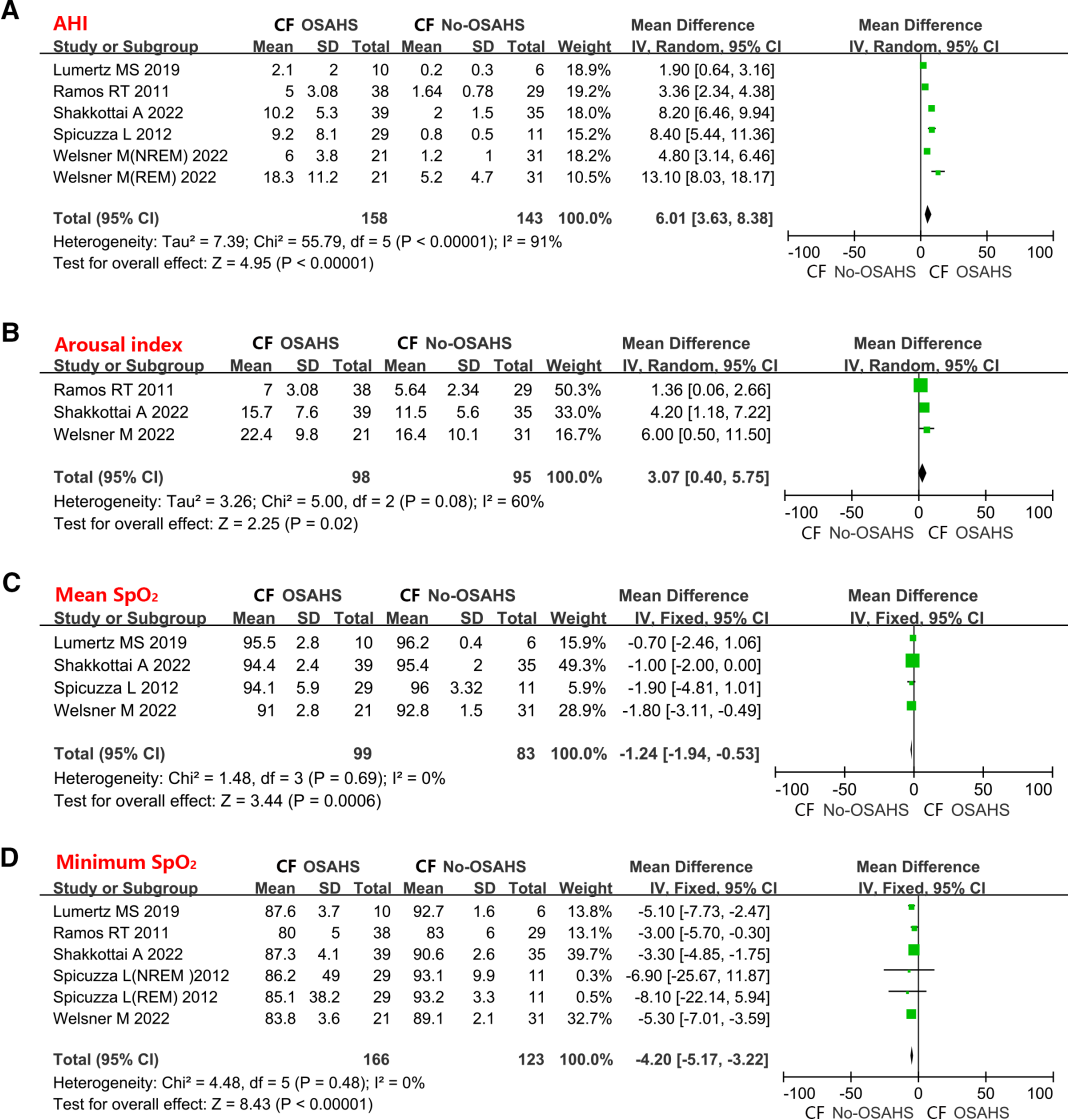
Effect of OSAHS on sleep indicators in patients with CF. (A) AHI, (B) Arousal index, (C) Mean oxygen saturation, (D) Minimum oxygen saturation. AHI = apnea-hypopnea index, CF = cystic fibrosis, CI = confidence interval, OSAHS = obstructive sleep apnea-hypopnea syndrome.

The AHI was markedly elevated in participants with both CF and OSAHS (MD: 6.01; 95% CI: 3.63–8.38; *I*^2^ = 91%; 6 studies) (Fig. 5A). The forest plot suggested that the AHI difference among the OSAHS-positive and OSAHS-negative groups was most pronounced during the REM stage (MD = 13.10). Additionally, the arousal index was markedly higher in the OSAHS-positive group (MD: 3.07; 95% CI: 0.40–5.75; *I*^2^ = 60%; 3 studies) (Fig. 5B). However, the mean SpO_2_ (MD: −1.24; 95% CI: −1.94–−0.53; *I*^2^ = 0%; 4 studies) (Fig. 5C) and minimum SpO_2_ (MD: -4.20; 95% CI: −5.17–−3.22; *I*^2^ = 0%; 6 studies) were markedly lower in the OSAHS-positive group (Fig. 5D). The difference in minimum SpO_2_ was particularly evident during the REM stage (MD = −8.10). The summary of the impact of OSAHS on sleep indexes in patients with CF is shown in Table 3.

**Table 3:** Differences in sleep indexes and lung functions between CF patients with negative OSAHS and positive OSAHS.

Indicators	n	Sample		MD (95% CI)	*P*_h_	*I*^2^ Value (%)	*P* Value between groups
		OSAHS-positive	OSAHS-negative				
AHI	6	158	143	6.01 [3.63–8.38]	< .001	91	< .001
Arousal index	3	98	95	3.07 [0.40–5.75]	.08	60	.02
Mean SpO_2_	4	99	83	1.24 [−1.94–−0.53]	.69	0	.001
Minimum SpO_2_	6	166	123	4.20 [−5.17–−3.59]	.48	0	< .001
FEV1, % predicted	4	70	69	7.63 [−14.37–−0.89]	.28	21	.03
FVC, % predicted	4	70	69	6.96 [−13.67–−0.25]	.27	24	.04

AHI = apnea-hypopnea index, CF = cystic fibrosis, CI = confidence interval, FEV1 = forced expiratory volume in 1 second, FVC = forced vital capacity, MD = mean difference, n = number of studies, OSAHS = obstructive sleep apnea-hypopnea syndrome, *P*_h_ = *P* for heterogeneity, SpO_2_ = peripheral oxygen saturation.

#### 3.4.4. Impact of OSAHS on pulmonary function in patients with CF

**Figure 6.**
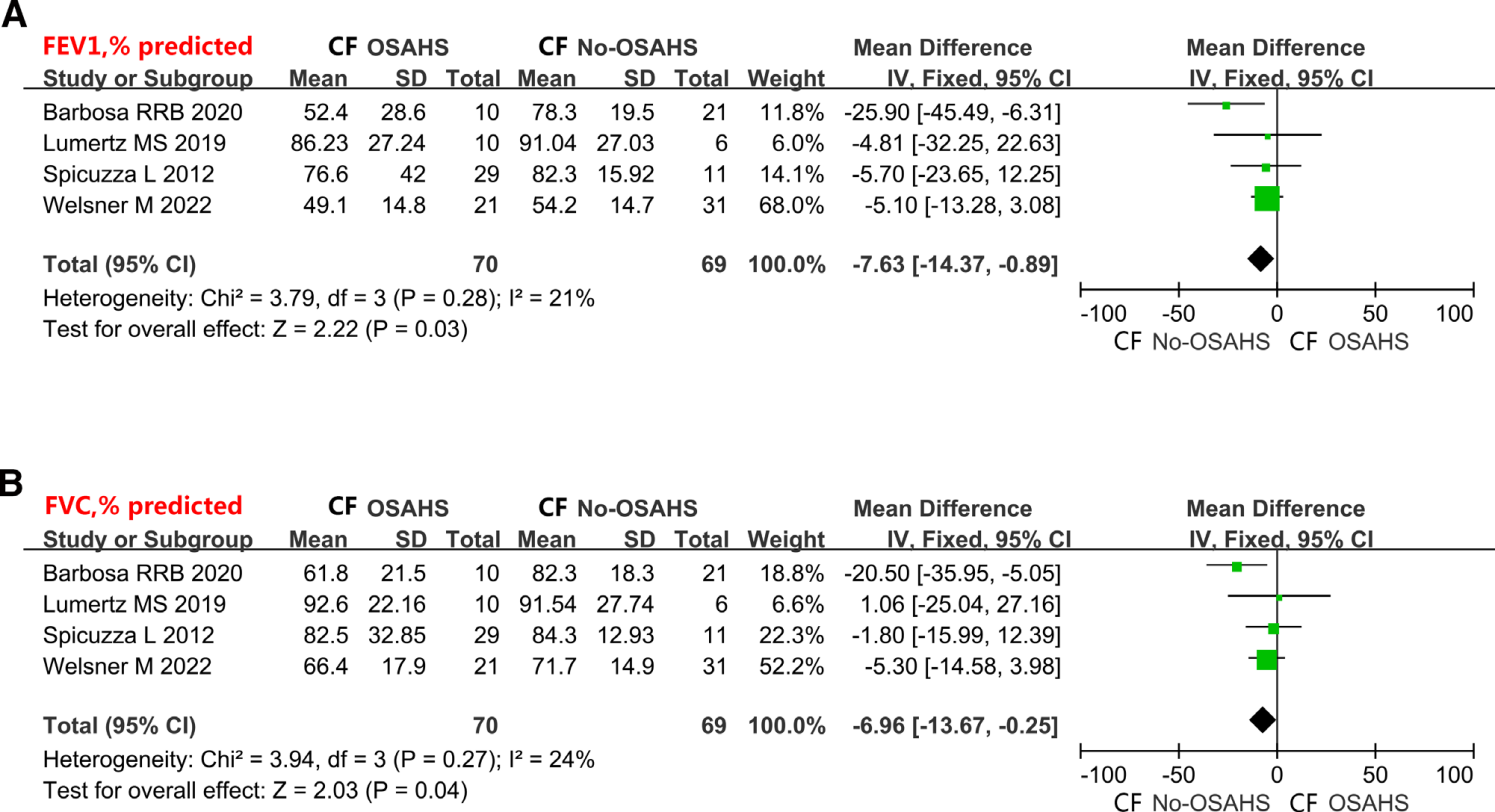
Effect of OSAHS on pulmonary function in patients with CF. (A) FEV1, % predicted, (B) FVC, % predicted. AHI = apnea-hypopnea index, CF = cystic fibrosis, CI = confidence interval, FEV1 = forced expiratory volume in 1 second, FVC = forced vital capacity, IV = instrumental variable, OSAHS = obstructive sleep apnea-hypopnea syndrome, SD = standard deviation.

Four studies provided data on FEV1, % predicted and FVC, % predicted in patients with CF. The meta-analysis suggested that FEV1, % predicted was lower in the OSAHS-positive group in comparison to the OSAHS-negative group (MD: −7.63; 95% CI: −14.37–−0.89; *I*^2^ = 21%; 4 studies) (Fig. 6A) (Table 3). Similarly, FVC, % predicted was also lower in the OSAHS-positive group in comparison to the OSAHS-negative group (MD: −6.96; 95% CI: −13.67–−0.25; *I*^2^ = 24%; 4 studies) (Fig. 6B) (Table 3).

## 4. Discussion

This systematic review and meta-analysis offer valuable insights into the clinical significance of OSAHS in individuals with CF. The study elucidates the prevalence of OSAHS in individuals with CF and examines how age influences its prevalence. Additionally, it compares differences in sleep monitoring parameters and pulmonary function between CF patients with and without OSAHS.

In the reviewed studies, the prevalence of OSAHS in individuals with CF varied from 32 to 79%. This considerable variation primarily stems from the differing diagnostic criteria for OSAHS. When the AHI cutoff is standardized to > 1/hour for pediatric OSAHS diagnosis, the prevalence is consistently high across studies, with nearly all reporting OSAHS prevalence above 50%. However, research has indicated that OSAHS prevalence in healthy children and adults is between 1.2% and 5.7%.^[36–38]^ In contrast, a pediatric cohort study involving symptomatic or clinically manifesting sleep disorders showed a prevalence of approximately 51%.^[39]^ This study also observed a high prevalence of OSAHS in adult individuals with CF, with both pediatric and adult groups displaying mean AHI values above the normal range. OSAHS appears to be a frequent complication in patients with CF. Previous literature reported a very low prevalence of OSAHS in adults with CF,^[25]^ while this study identified a markedly higher prevalence. It is well established that increasing age and weight gain are risk factors for OSAHS in the general population. Examination of data from the Canadian CF Registry revealed that the median survival age of individuals with CF has markedly elevated due to advancements in disease management, rising from 31.9 years in 1990 to 49.7 years in 2012.^[18]^ Between 1993 and 2018, the proportion of individuals classified as overweight or obese by BMI in the CF population also grew annually.^[10]^ In the adult CF population, increased life expectancy and weight gain may explain the higher prevalence of OSAHS found in this study compared to earlier reports. Moreover, some CFTR modulator drugs have known side effects of weight gain.^[19]^ As CFTR modulator therapy becomes more widespread among patients with CF, the prevalence of high BMI may rise, potentially increasing OSAHS prevalence.

The correlation between sleep disorders and the severity of CF, coupled with the influence of sleep apnea on the management of patients with CF, remains unclear.^[10]^ No direct causal phenomena have been identified in these studies, and the diversity of the research complicates determining the primary mechanisms by which CF induces OSAHS. The definition of OSAHS in pediatric patients remains a topic of debate. The American Academy of Sleep Medicine suggests an obstructive AHI of 1 per hour of sleep, but some pediatric investigations have employed a threshold of AHI > 2 per hour.^[40]^ Moreover, certain researchers have applied adult standards to patients with CF aged 12 years and older (AHI > 5 per hour). In this meta-analysis, only 1 author considered this criterion, and even when utilizing this threshold, they reported AHI values for those under and over 12 years of age.^[26]^ Ramos et al (2011) did not use hypopnea events and instead employed an obstructive apnea index of 1 per hour, finding OSAS in 56.7% of their study sample.^[9]^ Two other studies reported lower prevalence rates; however, it should be noted that both studies had very small sample sizes (10 participants each) and failed to specify their OSAHS definition criteria.^[23,41]^

The occurrence of OSAHS in healthy pediatric patients is linked to structural changes in the upper airway. In a randomized controlled trial utilizing an AHI ≥ 2 as the diagnostic criterion, researchers found that the prevalence of OSAHS in children with upper airway structural changes was 33%.^[40]^ This finding aligns with the investigation by Barbosa et al,^[5]^ who reported a prevalence of 32.3% for OSAHS in pediatric patients with CF using the same criteria. Spicuzza et al (2012)^[24]^ observed that the AHI was marginally elevated in the CF cohort relative to the control group, which lacked CF but exhibited upper airway anatomical irregularities. Consequently, OSAS seems to be prevalent in pediatric patients with CF, irrespective of alterations in the upper airway structure. These observations are consistent with the current study, where adults and children exhibited higher mean AHI values, especially during REM sleep (AHI = 16.32). The suboptimal sleep quality in patients with CF underscores the need for active screening for OSAHS.

This study also discovered that CF individuals with OSAHS had poorer pulmonary function, significant nocturnal hypoxemia, and microarousals. Another study involving both pediatric and adult patients with CF reported a correlation between FEV1% and AHI.^[42]^ Although data on the interaction between OSAHS and CF are limited, these findings indicate a bidirectional, vicious cycle association between OSAHS and the severity of CF disease in these patients.

Notably, nocturnal hypoxemia is a prevalent manifestation in patients with CF. The severity of pulmonary involvement in CF strongly correlates with the development of nocturnal hypoxemia.^[5,23,43,44]^ This meta-analysis suggests that CF patients with OSAHS endure more severe nocturnal hypoxemia. Detecting and correcting nocturnal hypoxemia markedly impacts the progression of CF and patient health. Chronic nocturnal hypoxemia can lead to reduced sleep quality, impaired glucose regulation, decreased quality of life, pulmonary hypertension, neurocognitive impairment, and daytime sleepiness.^[13,45,46]^ Hence, early PSG screening to detect nocturnal hypoxemia is crucial.

The prevalence of OSAHS in patients with CF may have multiple causes beyond lung disease severity. Most studies included individuals with normal or mildly impaired pulmonary function, evaluated during relatively stable periods. CF patients with OSAHS exhibited higher arousal indexes, indicating widespread sleep disruption among patients with CF. This sleep disruption is not solely due to exchange abnormalities; it is also associated with chronic lung disease factors such as nocturnal coughing and gastroesophageal reflux caused by OSAHS.^[47,48]^ Additionally, sleep may be impacted by time-consuming treatments, including manual chest physiotherapy, high-frequency chest wall oscillation vests, and oral or inhaled corticosteroid medications.^[49]^ These presleep treatments influence sleep both behaviorally and pharmacologically. Studies indicate that patients with respiratory disease on oral corticosteroids have an increased likelihood of respiratory sleep disorders, as glucocorticoids can cause cervical and centripetal fat deposition and myopathy.^[50,51]^ In patients with asthma, inhaled corticosteroids are also dose-dependently associated with OSAHS, regardless of asthma severity.^[52]^ CF impacts the whole respiratory system, resulting in nasal and lung diseases and impaired mucociliary clearance, which can lead to chronic sinusitis.^[53]^ Chronic sinusitis can narrow the upper airway, contributing to the pathophysiology of OSAHS by affecting nasal breathing. Questionnaire surveys and otolaryngological examinations reveal that upper airway changes due to nasal polyps and chronic infections in pediatric and adult patients with CF are closely linked to the development of OSAHS.^[54]^ The elevated prevalence of OSAHS among individuals with CF could be attributed to this finding. Furthermore, CF patients with OSAHS may also suffer from chronic pain, akin to other chronic pain conditions,^[55]^ although the precise mechanism by which pain disrupts sleep remains unclear. Two-thirds of pediatric patients with CF report recurrent pain episodes, primarily abdominal, but also musculoskeletal and joint pain.^[56]^ Decreased sleep quality caused by OSAHS can lead to a heightened perception of pain symptoms.^[57]^ Enhancing sleep quality may reduce pain in pediatric patients with CF, although this requires further investigation. A previous review outlined the prevalence of pain and sleep problems in pediatric patients with CF, highlighting the lack of studies linking the 2.^[57]^

In conclusion, evidence suggests that CF and OSAHS are mutually aggravating risk factors. One possible explanation lies in the exacerbation periods experienced by patients with CF, including recurrent coughing, epithelial and upper respiratory tract damage, and inflammation caused by neutrophil infiltration, which leads to increased peripheral oropharyngeal soft tissue and reduced airway diameter, resulting in airway obstruction during sleep. Conversely, severe sleep deprivation and oxidative stress caused by OSAHS increase the risk of acute CF exacerbations, leading to repeated worsening of the condition. Although the study by de Sousa LP et al^[22]^ reported a high prevalence of OSAHS in pediatric and adult individuals with CF, the present study includes more adult samples and analyzes the impact of OSAHS on sleep parameters and pulmonary function in patients with CF. This is significant in helping clinicians understand the interaction mechanism between CF and OSAHS.

The main limitations of this systematic review include potential confounding factors that might affect the AHI in patients with CF, such as CF-related diabetes, liver fibrosis, and portal hypertension, despite performing a subgroup analysis based on age. Another limitation is the variability in methodological criteria used across different pediatric OSAHS studies, complicating the inclusion of more data in the meta-analysis. Most of the included studies were retrospective, and only 4 were prospective. An important methodological consideration is the disparity between prospective and retrospective study designs. Prospective studies enrolled CF participants consecutively and performed PSG regardless of sleep-related symptoms, enabling unbiased ascertainment of OSAHS prevalence. Retrospective studies, by contrast, identified patients who had already undergone PSG, most of whom were referred due to suspected sleep-disordered breathing. These 2 populations are not directly comparable, and the selective inclusion of symptomatic patients in retrospective analyses likely inflates prevalence estimates. Only prospective studies with consecutive, unselected enrollment can determine the true global prevalence of OSAHS in the CF population, and future meta-analyses may benefit from stratifying or subgrouping studies by design type to quantify this bias. Most of the articles were observational studies from Europe, the United States, and Brazil, hindering the analysis of causal relationships. This may be a cause of the relatively high heterogeneity. To clarify the sources of heterogeneity, we conducted the subgroup and sensitivity analyses (i.e., removal of individual studies and observing any change in overall heterogeneity). However, in our meta-analysis, we failed to find the source of heterogeneity. Additionally, limited research has investigated the association between OSAHS and factors like nasal polyps, nutritional status, and other clinical factors, preventing clarification of the high prevalence of OSAHS in this population. Therefore, upcoming research should evaluate the relationship between OSAHS and clinical factors. For instance, to gain a better understanding of these associations: hospitalization, antibiotic use, nutritional status, and endoscopic assessment of the upper respiratory tract. Furthermore, studies employing experimental designs are necessary to establish causal relationships.

## 5. Conclusion

The meta-analysis revealed a high prevalence of OSAHS in both pediatric and adult individuals with CF. OSAHS may affect sleep monitoring parameters and pulmonary function in patients with CF, highlighting the significance of examining sleep disorders in this population. PSG screening offers an opportunity for early intervention of OSAHS in both pediatric and adult patients with CF.

## Acknowledgments

We thank Bullet Edits Limited for the linguistic editing and proofreading of the manuscript.

## Author contributions

**Conceptualization:** Hua Yu, Jiaqing Jiang.

**Dara curation:** Jun Zeng, Hua Yu, Jia Liu, Jiaqing Jiang Jia Li, Jie He.

**Methodology:** Jia Liu, Jia Li, Jie He.

**Formal analysis:** Jun Zeng, Jie He.

**Funding acquisition:** Jun Zeng, Jia Liu.

**Software:** Jun Zeng, Jie He.

**Validation:** Hua Yu, Jiaqing Jiang.

**Visualization:** Hua Yu.

**Writing – original draft:** Jun Zeng, Jie He.

**Writing – review & editing:** Hua Yu, Jia Liu, Jiaqing Jiang.








