# Supplementary material for: The prevalence of obstructive sleep apnea-hypopnea syndrome in patients with cystic fibrosis: An updated systematic review and meta-analysismeta-analysis
Source: Medicine (Baltimore). 2026 Jul 17;105(29):e49828. doi: 10.1097/MD.0000000000049828 (PMC13384674; doi:10.1097/MD.0000000000049828)
Supplement: Supplementary file 1 [file medi-105-e49828-s001.docx]

**Supplemental Material**

**Supplementary Table 1 Search strategies in each database---2025.2.6**

| **Database** | **Search strategies** | **Results** |
| --- | --- | --- |
| **PubMed** | #1 ("Sleep Apnea, Obstructive"[Mesh]) OR (sleep[Title/Abstract] OR Obstructive Sleep Apnea-Hypopnea Syndrome[Title/Abstract] OR Obstructive Sleep Apnea[Title/Abstract] OR Obstructive Sleep Apnea Syndrome[Title/Abstract] OR OSA[Title/Abstract] OR OSAHS[Title/Abstract] OR OSAS[Title/Abstract])---233,510  #2 ("Cystic Fibrosis"[Mesh]) OR (cystic fibrosis[Title/Abstract])---58,446  #3 #1 AND #2---355 | 355 |
| **Web of**  **Science** | #1 ((TS=(Obstructive Sleep Apnea-Hypopnea Syndrome)) OR TI=(sleep OR Obstructive Sleep Apnea-Hypopnea Syndrome OR Obstructive Sleep Apnea OR Obstructive Sleep Apnea Syndrome OR OSA OR OSAHS OR OSAS)) OR AB=(sleep OR Obstructive Sleep Apnea-Hypopnea Syndrome OR Obstructive Sleep Apnea OR Obstructive Sleep Apnea Syndrome OR OSA OR OSAHS OR OSAS)---187,526  #2 ((TS=(cystic fibrosis)) OR TI=(cystic fibrosis)) OR AB=(cystic fibrosis)---38,872  #3 #1 AND #2---289 | 289 |
| **Embase** | #1 'obstructive sleep apnea-hypopnea syndrome'/exp OR sleep:ti,ab,kw OR 'obstructive sleep apnea-hypopnea syndrome':ti,ab,kw OR 'obstructive sleep apnea':ti,ab,kw OR 'obstructive sleep apnea syndrome':ti,ab,kw OR osa:ti,ab,kw OR osahs:ti,ab,kw OR osas:ti,ab,kw---356,637  #2'cystic fibrosis'/exp OR 'cystic fibrosis':ti,ab,kw---105,402  #3 #1 AND #2---893 | 893 |
| **Cochrane library** | #1 (sleep OR Obstructive Sleep Apnea-Hypopnea Syndrome OR Obstructive Sleep Apnea OR Obstructive Sleep Apnea Syndrome OR OSA OR OSAHS OR OSAS):ti,ab,kw (Word variations have been searched)---55,644  #2 MeSH descriptor: [cystic fibrosis] explode all trees---2,371  #3 (cystic fibrosis):ti,ab,kw (Word variations have been searched)---6,459  #4 #2 OR #3---2,371  #5 #1 AND #4---32 | 32 |
| **CNKI** | #1 (SU=Obstructive Sleep Apnea-Hypopnea Syndrome) OR (TKA=sleep OR Obstructive Sleep Apnea-Hypopnea Syndrome OR Obstructive Sleep Apnea OR Obstructive Sleep Apnea Syndrome OR OSA OR OSAHS OR OSAS)---34,746  #2 (SU=cystic fibrosis) OR (TKA=cystic fibrosis)---57,338  #3 #1 AND #2---38 | 38 |
