# Supplementary material for: The prevalence of obstructive sleep apnea-hypopnea syndrome in patients with cystic fibrosis: An updated systematic review and meta-analysismeta-analysis
Source: Medicine (Baltimore). 2026 Jul 17;105(29):e49828. doi: 10.1097/MD.0000000000049828 (PMC13384674; doi:10.1097/MD.0000000000049828)
Supplement: Supplementary file 2 [file medi-105-e49828-s002.docx]

| Supplementary Table 2 | | | | | | | | | | |
| --- | --- | --- | --- | --- | --- | --- | --- | --- | --- | --- |
| Critical evaluation of methodological quality | | | | | | | | | | |
| Studies | Year | Q1 | Q2 | Q3 | Q4 | Q5 | Q6 | Q7 | Q8 | Total |
| Maqsood A | 2024 | Y | Y | Y | Y | Y | S | Y | Y | 8 |
| Shakkottai A | 2024 | Y | Y | Y | Y | Y | S | Y | Y | 8 |
| Vezir D | 2023 | U | Y | Y | Y | Y | S | Y | Y | 7 |
| Shakkottai A | 2022 | U | Y | Y | Y | Y | S | Y | U | 6 |
| Welsner M | 2022 | Y | Y | Y | Y | Y | S | Y | Y | 8 |
| Barbosa RRB | 2020 | Y | Y | Y | Y | U | n/a | Y | Y | 6 |
| Shakkottai A | 2020 | Y | Y | Y | Y | Y | S | Y | Y | 8 |
| Isaiah A | 2019 | Y | Y | Y | Y | Y | S | Y | Y | 8 |
| Lumertz MS | 2019 | Y | Y | Y | Y | U | n/a | Y | Y | 6 |
| Waters KA | 2017 | U | Y | Y | Y | Y | S | Y | Y | 7 |
| Veronezi J | 2015 | Y | Y | Y | Y | Y | S | Y | Y | 8 |
| Perin C | 2012 | Y | Y | Y | Y | Y | S | Y | Y | 8 |
| Spicuzza L | 2012 | U | Y | Y | Y | N | N | Y | U | 4 |
| Ramos RT | 2011 | Y | Y | Y | Y | U | n/a | Y | Y | 6 |
| de Castro-Silva C | 2009 | Y | Y | Y | Y | Y | S | Y | Y | 8 |
| Y: yes; N: no; U: unclear; n/a: not applicable. | | | | | | | | | | |
