# Supplementary figures and images for: The prevalence of obstructive sleep apnea-hypopnea syndrome in patients with cystic fibrosis: An updated systematic review and meta-analysismeta-analysis
Source: Medicine (Baltimore). 2026 Jul 17;105(29):e49828. doi: 10.1097/MD.0000000000049828 (PMC13384674; doi:10.1097/MD.0000000000049828)

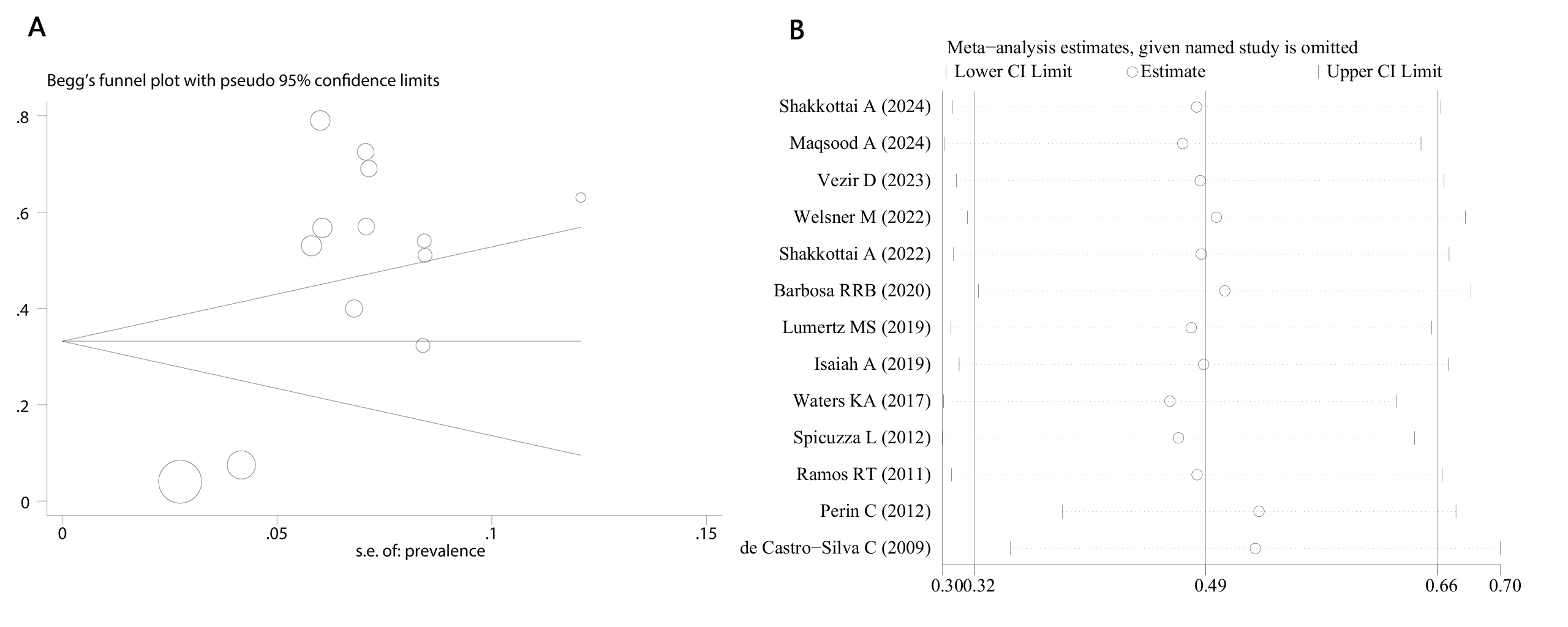

Supplement: Supplementary file 3 [file medi-105-e49828-s003.tif]

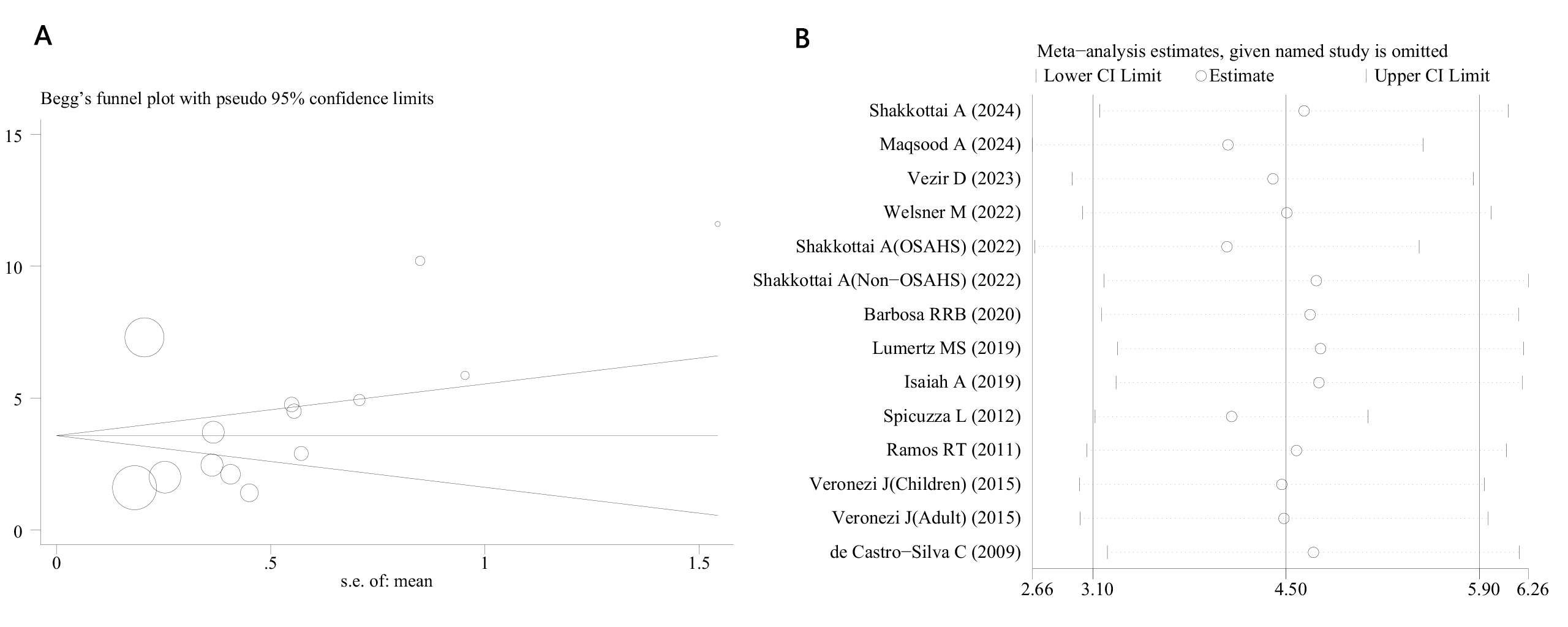

Supplement: Supplementary file 4 [file medi-105-e49828-s004.tif]
